# Regorafenib with immunotherapy versus regorafenib alone as second‐line treatment for hepatocellular carcinoma: A multicenter real‐world study

**DOI:** 10.1002/cam4.7236

**Published:** 2024-05-08

**Authors:** Liang Qiao, Wei He, Guoying Wang, Huanwei Chen, Fuxi Huang, Bo Zhang, Yuxiong Qiu, Shaoru Liu, Zhenkun Huang, Yichuan Yuan, Jiliang Qiu, Yunfei Yuan, Binkui Li

**Affiliations:** ^1^ State Key Laboratory of Oncology in South China, Guangdong Provincial Clinical Research Center for Cancer Sun Yat‐sen University Cancer Centre Guangzhou China; ^2^ Department of Liver Surgery Sun Yat‐Sen University Cancer Center Guangzhou China; ^3^ Department of Hepatobiliary Surgery the First Affiliated Hospital of Guangzhou Medical University Guangzhou China; ^4^ Department of Hepatic Surgery and Liver Transplantation The Third Affiliated Hospital of Sun Yat‐Sen University Guangzhou China; ^5^ Department of Hepatopancreatic Surgery the First People's Hospital of Foshan Foshan China; ^6^ Department of Oncology Guangzhou Panyu Central Hospital Guangzhou China; ^7^ Department of Internal Medicine Guangdong Panyu District He Xian Memorial Hospital Guangzhou China

**Keywords:** hepatocellular carcinoma, immune checkpoint inhibitor, multicenter, regorafenib, second‐line therapy

## Abstract

**Introduction:**

Regorafenib remains the standard and widely used second‐line strategy for advanced hepatocellular carcinoma (HCC). There is still a lack of large‐scale multicenter real‐world evidence concerning the concurrent use of regorafenib with immune checkpoint inhibitors (ICI). This study aims to evaluate whether combining regorafenib with ICI provides greater clinical benefit than regorafenib monotherapy as second‐line therapy for advanced HCC under real‐world circumstances.

**Patients and Methods:**

The study included 208 patients from five medical facilities. One hundred forty‐three patients received regorafenib plus ICI combination therapy, while 65 patients received regorafenib monotherapy. Propensity score matching (PSM) analysis was employed.

**Results:**

The regorafenib plus ICI group demonstrated significantly higher objective response rate (24.3% vs. 10.3%, after PSM, *p* = 0.030) and disease control rate (79.4% vs. 50.0%, after PSM, *p* < 0.001) compared to the regorafenib monotherapy group based on mRECIST criteria. Median progression‐free survival (7.9 vs. 3.2 months, after PSM, *p* < 0.001) and overall survival (25.6 vs. 16.4 months, *p* = 0.010, after PSM) were also considerably longer in the regorafenib plus ICI group. The incidence of Grades 3–4 treatment‐related adverse events (TRAEs) was marginally greater in the regorafenib plus ICI group than in the regorafenib group (23.8% vs. 20.0%, *p* = 0.546). Notably, there were no instances of treatment‐related mortality or emergence of new TRAEs in any treatment group.

**Conclusion:**

The combination of regorafenib and ICI shows potential as a viable second‐line treatment for advanced HCC, exhibiting favorable efficacy while maintaining a tolerable safety profile in contrast to regorafenib monotherapy.

## Graphical abstract

Regorafenib+ICI was more effective than regorafenib in second line for advanced HCC. Combining with ICI did not yield a significant increase in TRAEs.

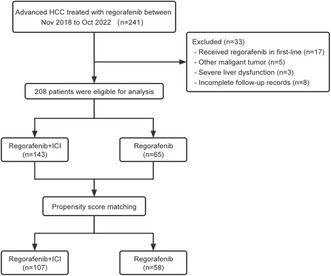

## 1 INTRODUCTION

Hepatocellular carcinoma (HCC) continues to be a significant public health concern, ranking as the third leading cause of cancer‐related mortality globally.^1^ HCC is often asymptomatic at the early stage, leading to the majority of patients being diagnosed at the advanced stage with a poor prognosis.^2^ Systemic therapy, represented by multikinase inhibitors sorafenib and lenvatinib, has been widely recognized as the recommended treatment for advanced HCC.^3^, ^4^ The advent of immune checkpoint inhibitors (ICI) significantly altered the therapeutic landscape for cancer. The combination of atezolizumab, a programmed death ligand 1 (PD‐L1) inhibitor, and bevacizumab, a monoclonal antibody against vascular endothelial growth factor (VEGF), demonstrated significantly greater efficacy over sorafenib, highlighting the enormous potential of combination therapy.^5^ Despite these advances in systemic therapy, achieving complete response in HCC remains rare, necessitating a switch to second‐line treatment upon disease progression.

At present, multikinase inhibitors regorafenib, cabozantinib, and ramucirumab have been approved for patients with advanced HCC who progressed on sorafenib by the US Food and Drug Administration (US FDA). The median overall survival (OS) of monotherapy in previous Phase 3 trials ranged from 9.2 to 10.6 months.^6^, ^7^, ^8^ The efficacy of monotherapy approaches for advanced HCC in second‐line scenarios appears to be limited, highlighting the urgent demand for more effective second‐line systemic therapies for HCC.

Regorafenib is an oral multikinase inhibitor with a broader spectrum of inhibited targets associated with angiogenesis, tumor proliferation, spread, and metastasis.^9^ Regorafenib can normalize tumor vasculature and alleviate immune suppression in the tumor microenvironment, thereby enhancing the antitumor efficacy when combined with ICI.^10^, ^11^, ^12^, ^13^, ^14^ In Phase Ib study, the combination of regorafenib and pembrolizumab (a PD‐1 inhibitor) demonstrated significant antitumor activity and tolerable safety as the first‐line therapy for advanced HCC, achieving a disease control rate (DCR) of 88%.^15^ Several Phase I/II clinical trials are in progress, investigating regorafenib with ICI in both first‐line and second‐line settings. Moreover, considering that multikinase inhibitors sorafenib and lenvatinib both have been widely used in first‐line clinical practice and their combinations with ICI have also been extensively explored,^16^ it is important to evaluate the second‐line treatment profile in real‐world scenarios. In this case, we conducted this multicenter, real‐world, retrospective study to evaluate the safety and efficacy of the combination of regorafenib with ICI compared to regorafenib monotherapy.

## 2 PATIENTS AND METHODS

### 2.1 Patients

This study included patients with advanced HCC who received regorafenib plus ICI or regorafenib monotherapy after disease progression between November 2018 and October 2022 at five medical centers in China.

This study was carried out in adherence to the Declaration of Helsinki and received approval from the ethics committee of all participating hospitals (approval number: B2023‐223‐01). The requirement for informed consent was waived. In addition, this study has been registered with the unique identification number: ChiCTR2400079560, https://www.chictr.org.cn/showproj.html?proj=214883.

Patients were included according to the following criteria: (1) HCC was diagnosed by histology or clinically confirmed in accordance with international guidelines^17^, ^18^; (2) age over 18 years old; (3) received at least one cycle of regorafenib plus ICI or regorafenib monotherapy subsequent to disease progression on first‐line systemic treatment; (4) received either sorafenib/lenvatinib monotherapy or sorafenib/lenvatinib combined with ICI as first‐line systemic treatment; (5) had at least one measurable lesion by modified Response Evaluation Criteria in Solid Tumors for HCC (mRECIST).^19^

Exclusion criteria were applied to patients who (1) had a concurrent malignant tumor other than HCC; (2) had severe medical comorbidities including cardiac, pulmonary, or renal dysfunction; (3) had incomplete follow‐up records.

### 2.2 Data collection

Baseline characteristics were retrieved from medical record systems within 30 days prior to the initiation of regorafenib treatment. If multiple data points were accessible, the nearest one to the regorafenib start date was chosen. Clinical, laboratory, and radiological data were obtained from medical record systems, including age, gender, BCLC stage, ECOG performance status, hepatitis B surface antigen (HBsAg), Child‐Pugh class, alpha‐fetoprotein (AFP) level, tumor size, tumor number, extrahepatic metastasis, previous treatment procedures, biochemical indices, and so on. All dynamic computed tomography (CT) and magnetic resonance (MR) analyses were based on independent assessments by two experienced radiologists.

### 2.3 Treatments and follow‐up

Regorafenib was initially administered orally once daily at a dosage of 80–160 mg depending on patient's tolerance, for the first 3 weeks of each 4‐week cycle. Treatment interruptions and dose reductions were allowed to manage toxicity. ICIs were prescribed intravenously to 143 patients in regorafenib plus ICI group every 3 weeks according to the standard drug instructions, including atezolizumab, camrelizumab, durvalumab, pembrolizumab, sintilimab, tislelizumab, and toripalimab. Follow‐up was conducted every 3–6 weeks until loss to follow‐up or death. Treatment continued until disease progression as defined by mRECIST, clinical progression (defined as an ECOG performance score ≥3 or symptomatic deterioration), death, or unacceptable toxicity. This study was censored on January 31, 2023.

### 2.4 Outcomes

Tumor responses were assessed based on CT or MR according to mRECIST.^19^ The objective response rate (ORR) was defined as the proportion of patients achieving complete response (CR) and partial response (PR). DCR was defined as the proportion of patients with CR, PR, and stable disease (SD). Progression‐free survival (PFS) was defined as the time from the initiation of regorafenib until the date when tumor progression or death was confirmed. OS was defined as the time from the initiation of regorafenib until death.^8^ Treatment‐related adverse events (TRAEs) were recorded according to the National Cancer Institute Common Terminology Criteria for Adverse Events (NCI‐CTCAE), version 5.0.^20^

### 2.5 Statistical analysis

Quantitative data were presented as median value (interquartile range [IQR]), and categorical data was expressed as number (percentage). The chi‐squared test or Fisher's exact test was used to compare categorical data. Independent‐sample *t*‐test or Mann–Whitney *U*‐test was used to compare quantitative data (baseline characteristics). Propensity score matching (PSM) with a ratio of 1:2 was performed using nearest neighbor algorithm.^21^ Survival curves were analyzed by the Kaplan–Meier method using the log‐rank test. Multivariate Cox regression analysis was performed to determine the independent factors significantly associated with PFS and OS. A backward stepwise regression method was employed to select the best combination of variables from the univariate analysis for the multivariate analysis model. In subgroup analysis, the Cox regression model was applied to compare PFS and OS between the two groups across different variables. *p* < 0.05 was considered statistically significant. All statistical analyses were performed using R version 4.2.2.

## 3 RESULTS

### 3.1 Study population and baseline characteristics

**FIGURE 1:**
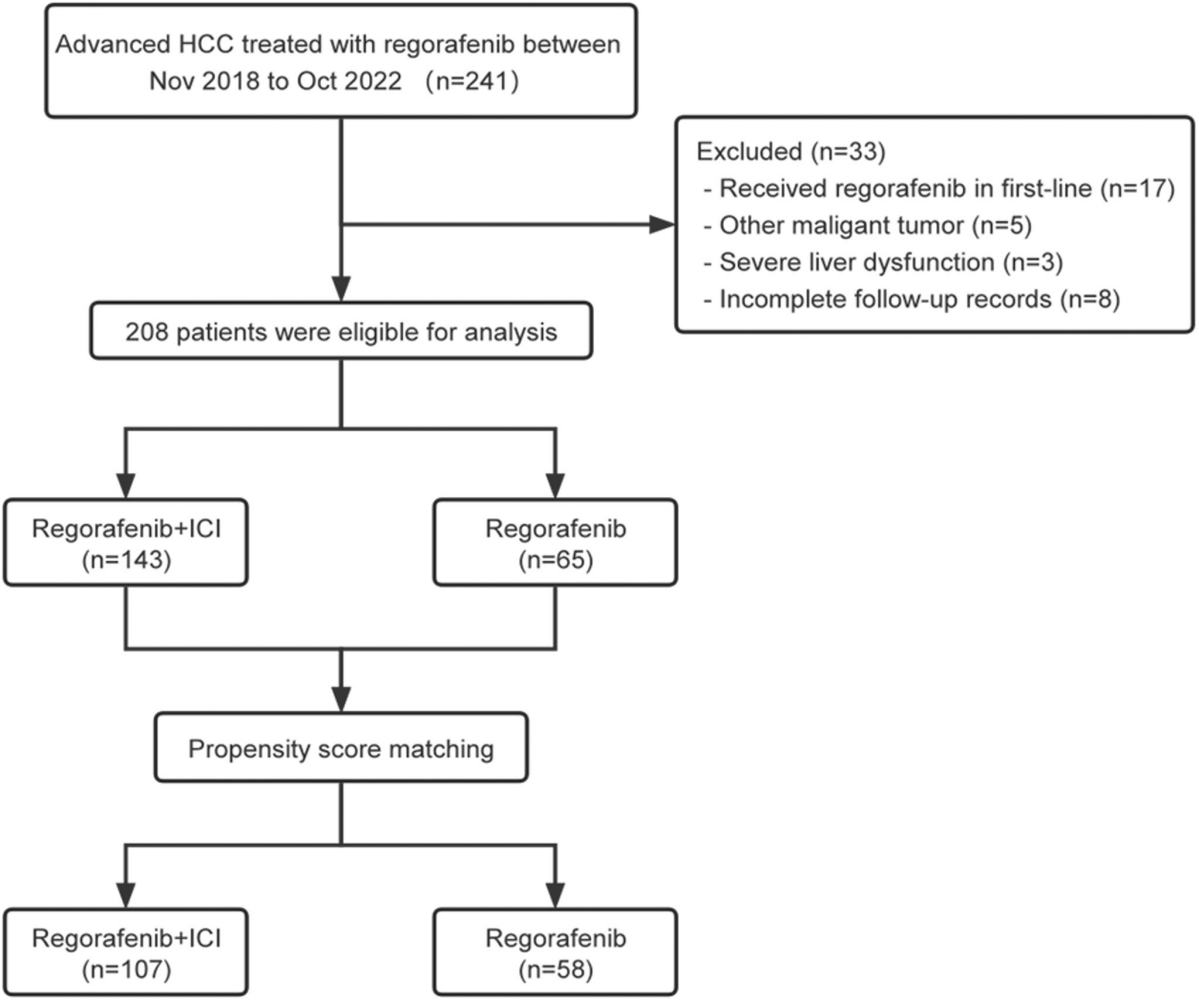
Flowchart of patient screening. HCC, hepatocellular carcinoma; ICI, immune checkpoint inhibitors.

A total of 208 patients were included in the final analysis (Figure 1). One hundred forty‐three patients received regorafenib plus ICI combination therapy, and 65 patients received regorafenib monotherapy. Baseline characteristics revealed imbalances between groups, mainly in terms of BCLC stage, first‐line treatment profile, and regorafenib initial dose. Specifically, the regorafenib plus ICI group had the worse BCLC stage (*p* = 0.021) and a higher proportion of patients who received ICI in the first‐line treatment (*p* < 0.001) compared with the regorafenib monotherapy group. After PSM, no statistically significant difference was observed between the two groups (Table 1).

**TABLE 1:** Baseline characteristics of patients at initiation of second‐line therapy before and after PSM.

Characteristic	Before PSM		*p* value	After PSM		*p* value
	Regorafenib + ICI	Regorafenib		Regorafenib + ICI	Regorafenib	
	(*n* = 143)	(*n* = 65)		(*n* = 107)	(*n* = 58)	
Sex, male	122 (85.3)	57 (87.7)	0.646	89 (83.2)	51 (87.9)	0.416
Age, years	52 (44–61)	51 (43–63)	0.554	53 (44–61)	52 (44–61)	0.893
ECOG performance status						
0	101 (70.6)	47 (72.3)	0.804	74 (69.2)	42 (72.4)	0.662
1	42 (29.6)	18 (27.7)		33 (30.8)	16 (27.6)	
HBsAg‐positive	125 (87.4)	59 (90.8)	0.482	97 (90.7)	54 (93.1)	0.772
Antiviral drugs						
Entecavir	93 (65.0)	45 (69.2)	0.784	73 (68.2)	42 (72.4)	0.728
Tenofovir	32 (22.4)	14 (21.5)		24 (22.4)	12 (20.7)	
Child‐Pugh class						
A	123 (86.0)	58 (89.2)	0.522	94 (87.9)	51 (87.9)	0.988
B	20 (14.0)	7 (10.8)		13 (12.1)	7 (12.1)	
BCLC stage						
B	32 (22.4)	26 (40.0)	0.013*	32 (29.9)	19 (32.8)	0.705
C	106 (74.1)	39 (60.0)		75 (70.1)	39 (67.2)	
Tumor size >5 cm	64 (44.8)	31 (47.7)	0.693	48 (44.9)	28 (48.3)	0.674
Tumor number >3	88 (61.5)	48 (73.8)	0.084	71 (66.4)	42 (72.4)	0.424
Macrovascular invasion	47 (32.9)	18 (27.7)	0.437	36 (33.6)	18 (58.1)	0.733
Extrahepatic metastasis	82 (57.3)	30 (46.2)	0.120	56 (52.3)	30 (51.7)	0.940
ALT (U/L)	35.1 (23.7–50.7)	36.1 (25.4–59.6)	0.529	35.3 (24.4–47.9)	36.7 (25.7–64.4)	0.358
Total bilirubin (μmol/L)	15.1 (11.1–19.9)	15.3 (10.2–20.4)	0.833	14.7 (11.0–19.0)	14.7 (9.9–20.4)	0.917
Albumin (g/L)	41.3 (36.4–44.5)	40.3 (37.3–44.0)	0.826	41.6 (37.0–44.5)	41.1 (37.3–44.2)	0.711
ALBI grade						
Grade 1	88 (61.5)	38 (58.5)	0.596	67 (62.6)	36 (62.1)	>0.999
Grade 2	52 (36.4)	27 (41.5)		39 (36.4)	22 (37.9)	
Grade 3	3 (2.1)	0 (0)		1 (0.9)	0 (0)	
AFP (ng/mL)	333 (14.8–6526)	990.0 (50.6–7097.0)	0.261	281 (12.2–5281.0)	1717.0 (108.9–9088.0)	0.074
PIVKA‐II (mAU/ml)	2060.2 (246.3–10650.0)	2542.4 (491.0–10571.2)	0.565	3799.1 (676.1–12523.2)	3545.5 (1049.6–11732.2)	0.733
First‐line multikinase inhibitor						
Sorafenib	61 (42.7)	43 (66.2)	0.002*	56 (52.3)	36 (62.1)	0.229
Lenvatinib	82 (57.3)	22 (33.8)		51 (47.7)	22 (37.9)	
First‐line treatment with ICI	93 (65.0)	25 (38.4)	<0.001*	60 (56.0)	25 (43.1)	0.111
Duration of first‐line treatment, months	7.1 (3.2–11.7)	6.5 (3.2–10.7)	0.793	6.4 (3.3–10.8)	7.0 (3.3–11.5)	0.70
Regorafenib initial dose						
80 mg	63 (44.1)	21 (32.3)	0.016*	43 (40.2)	21 (36.2)	0.123
120 mg	39 (27.3)	12 (18.5)		30 (28.0)	10 (17.2)	
160 mg	41 (28.7)	32 (49.2)		34 (31.8)	27 (46.6)	
Locoregional treatment in second‐line treatment	72 (50.3)	28 (43.1)	0.331	58 (54.2)	26 (44.8)	0.250

*Note*: Data are *n* (%) or median (IQR), unless otherwise specified. Statistical significance indicated in bold (*p*<0.05).

Abbreviations: ALT, alanine transaminase; ALBI, albumin–bilirubin; AFP, α‐fetoprotein; BCLC, Barcelona clinic liver cancer; ECOG, Eastern Cooperative Oncology Group; HBsAg, hepatitis B surface antigen; ICI, immune checkpoint inhibitors; PIVKA‐II, protein induced by vitamin K absence or antagonist‐II; PSM, propensity score matching.

### 3.2 Tumor response

The regorafenib plus ICI group demonstrated significantly higher ORR (23.8% vs. 9.2%, before PSM, *p* = 0.014; 24.3% vs. 10.3%, after PSM, *p* = 0.030) and DCR (74.8% vs. 53.8%, before PSM, *P* = 0.003; 79.4% vs. 50.0%, after PSM, *P* < 0.001) compared to the regorafenib monotherapy group based on mRECIST. In detail, two patients in the regorafenib plus ICI group versus no patients in the regorafenib group achieved CR before PSM. The regorafenib plus ICI group exhibited a higher proportion of PR and rather less disease progression (PD) compared to the regorafenib monotherapy group, both before and after PSM (Table 2).

**TABLE 2:** Tumor response before and after PSM by mRECIST.

	Before PSM		*p* value	After PSM		*p* value
	Regorafenib + ICI	Regorafenib		Regorafenib + ICI	Regorafenib	
	(*n* = 143)	(*n* = 65)		(*n* = 107)	(*n* = 58)	
Best overall response						
Complete response (CR)	2 (1.4)	0 (0)	1	1 (1.0)	0 (0)	1
Partial response (PR)	32 (22.4)	6 (9.2)	0.023*	25 (23.4)	6 (10.3)	0.041*
Stable disease (SD)	73 (51.0)	29 (44.6)	0.390	59 (55.1)	23 (39.7)	0.058
Progressive disease (PD)	36 (25.2)	28 (43.1)	0.010*	22 (20.6)	27 (46.6)	0.001*
Not evaluable	0 (0)	2 (3.1)	0.097	0 (0)	2 (3.4)	0.122
Objective response	34 (23.8)	6 (9.2)	0.014*	26 (24.3)	6 (10.3)	0.030*
Disease control	107 (74.8)	35 (53.8)	0.003*	85 (79.4)	29 (50.0)	<0.001*

*Note*: Data are presented as number (percentage). Objective response = CR + PR; disease control = CR + PR + SD. Statistical significance indicated in bold (*p*<0.05).

Abbreviations: ICI, immune checkpoint inhibitors; mRESICT, modified response evaluation criteria in solid tumors; PSM, propensity score matching.

### 3.3 Survival analysis

The median follow‐up duration was 13.5 months (95% CI: 12.6–15.6 months) in the regorafenib plus ICI group and 14.8 months (95% CI: 12.2–18.8) in the regorafenib monotherapy group before PSM and remained similar after PSM.

**FIGURE 2:**
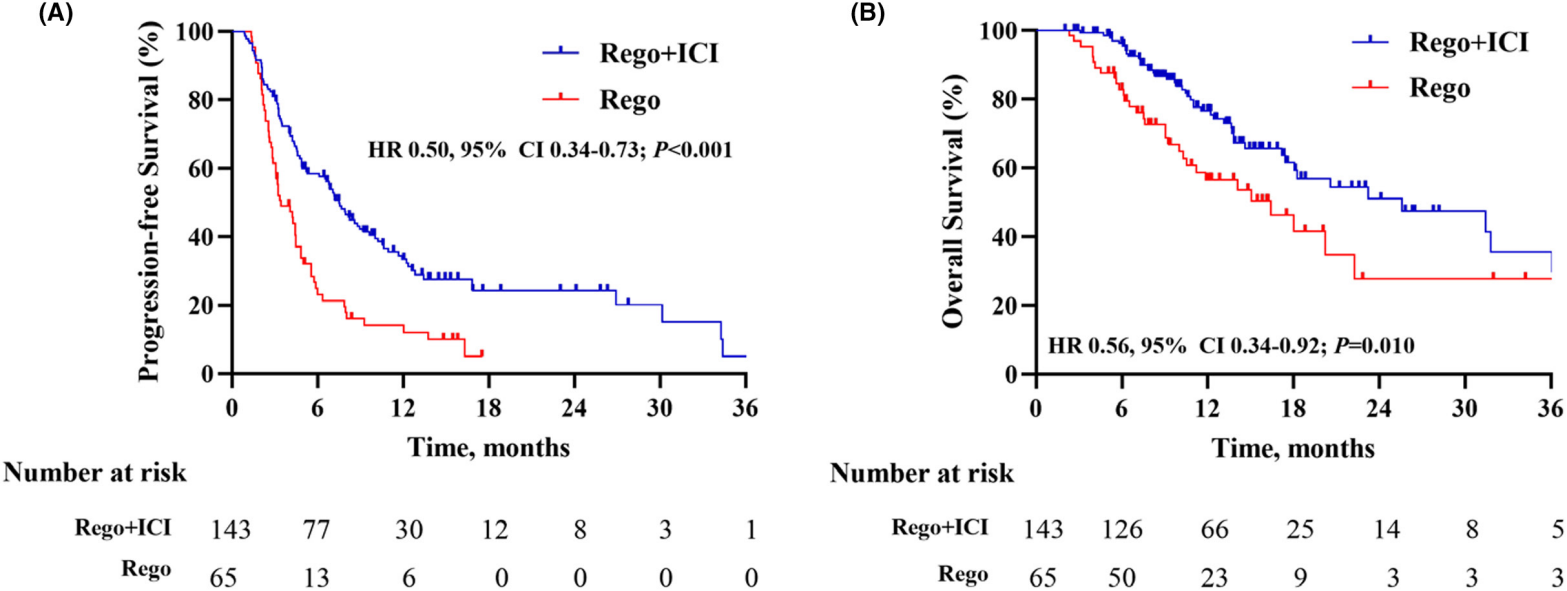
Before propensity score matching, Kaplan–Meier analysis of progression‐free survival (A) and overall survival (B) between regorafenib plus immune checkpoint inhibitors group (Rego + ICI) and regorafenib monotherapy group (Rego). CI, confidence interval; HR, hazard ratio.

Before PSM, the median PFS was 7.5 months (95% CI: 6.5–9.6) in the regorafenib plus ICI group and 3.4 months (95% CI: 3.0–4.9) in the regorafenib monotherapy group (*p* < 0.001, Figure 2A). The median OS was 25.6 months (95% CI: 18.1‐NA) in the regorafenib plus ICI group and 16.4 months (95% CI: 10.6‐NA) in the regorafenib monotherapy group (*P* = 0.010, Figure 2B).

**FIGURE 3:**
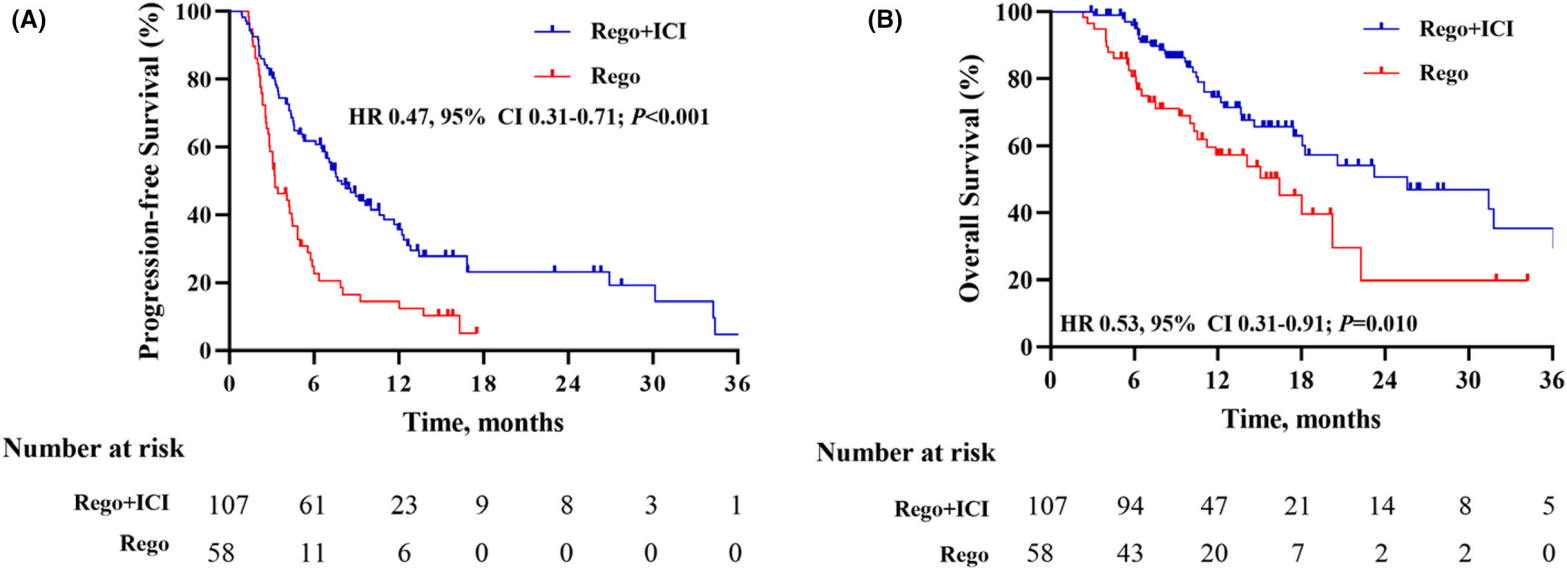
After propensity score matching, Kaplan–Meier analysis of progression‐free survival (A) and overall survival (B) between regorafenib plus immune checkpoint inhibitors group (Rego+ICI) and regorafenib monotherapy group (Rego). CI, confidence interval; HR, hazard ratio.

After PSM, the regorafenib plus ICI group showed a similarly significant improvement in both PFS (7.9 months, 95% CI: 6.6–11.7) and OS (25.6 months, 95% CI: 18.1‐NA), compared to the regorafenib monotherapy group's median PFS (3.2 months, 95% CI: 2.8–4.9; P < 0.001) and OS (16.4 months, 95% CI: 11.3‐NA; P = 0.010) (Figure 3 A‐B).

In cohort before PSM, multivariate analysis showed that presence of macrovascular invasion (HR = 1.45, 95% CI: 1.02–2.06, *p* = 0.039), first‐line treatment with lenvatinib (HR = 2.27, 95% CI: 1.36–3.78, *p* = 0.002) and regorafenib plus ICI treatment (HR = 0.43, 95% CI: 0.31–0.61, *p* < 0.001) were independent prognostic factors for PFS (Table 3). Regorafenib plus ICI treatment (HR = 0.55, 95% CI: 0.35–0.87, *p* = 0.01) was associated with OS in multivariate analysis (Table 4).

**TABLE 3:** Univariate and multivariate analyses of influencing factors for progression‐free survival.

Characteristic	Univariable			Multivariable		
	HR	95% CI	*p* value	HR	95% CI	*p* value
Sex						
Male/female	0.78	0.49, 1.25	0.308			
Age group						
<65 years/≥65 years	1.06	0.70, 1.60	0.776			
ECOG performance status						
0/1	0.91	0.64, 1.29	0.597			
HBsAg‐positive						
No/yes	1.03	0.59, 1.79	0.911			
Child‐Pugh class						
A/B	1.12	0.68, 1.84	0.65	1.77	0.98, 3.22	0.06
BCLC stage						
B/C	1.09	0.77, 1.54	0.632			
Macrovascular invasion						
No/yes	1.34	0.95, 1.89	0.093	1.45	1.02, 2.06	0.039*
Extrahepatic metastasis						
No/yes	1.05	0.76, 1.45	0.773	1.38	0.98, 1.93	0.063
ALBI grade						
1	—	—		—	—	
2	1.13	0.81, 1.58	0.455	0.91	0.62, 1.32	0.616
3	0.27	0.04, 1.91	0.188	0.18	0.02, 1.39	0.1
AFP						
<400 ng/mL/≥400 ng/mL	1.5	1.09, 2.06	0.013*			
First‐line multikinase inhibitor						
Sorafenib/lenvatinib	1.56	1.13, 2.17	0.007*	2.27	1.36, 3.78	0.002*
First‐line immune checkpoint inhibitor						
No/yes	1.12	0.81, 1.56	0.477	0.67	0.40, 1.12	0.123
Regorafenib initial dose						
80 mg	—	—				
120 mg	0.8	0.53, 1.22	0.303			
160 mg	0.98	0.68, 1.41	0.919			
Locoregional treatment in second‐line treatment						
No/yes	0.95	0.69, 1.30	0.749			
Group						
Regorafenib/regorafenib+ICI	0.48	0.34, 0.67	<0.001*	0.43	0.31, 0.61	<0.001*

*Note:* Statistical significance indicated in bold (*p*<0.05).

Abbreviations: ALBI, albumin–bilirubin; AFP, α‐fetoprotein; BCLC, Barcelona Clinic Liver Cancer; CI, confidence interval; ECOG, Eastern Cooperative Oncology Group; HR, hazard ratio; HBsAg, hepatitis B surface antigen; ICI, immune checkpoint inhibitors.

**TABLE 4:** Univariate and multivariate analyses of influencing factors for overall survival.

Characteristic	Univariable			Multivariable		
	HR	95% CI	*p* value	HR	95% CI	*p* value
Sex						
Male/female	0.59	0.29, 1.19	0.142	0.61	0.30, 1.24	0.172
Age group						
<65 years/≥65 years	1.18	0.67, 2.09	0.559			
ECOG performance status						
0/1	0.98	0.61, 1.58	0.924			
HBsAg‐positive						
No/yes	1.5	0.55, 4.12	0.428			
Child‐Pugh class						
A/B	1.25	0.62, 2.52	0.535			
BCLC stage						
B/C	1.17	0.71, 1.94	0.535			
Macrovascular invasion						
No/yes	1.49	0.91, 2.44	0.116	1.49	0.91, 2.45	0.113
Extrahepatic metastasis						
No/yes	1.27	0.80, 2.02	0.32			
ALBI grade						
1	—	—				
2	0.89	0.54, 1.45	0.628			
3	0.93	0.13, 6.75	0.941			
AFP						
<400 ng/mL/≥400 ng/mL	1.45	0.92, 2.29	0.107			
First‐line multikinase inhibitor						
Sorafenib/lenvatinib	0.96	0.59, 1.56	0.863			
First‐line immune checkpoint inhibitor						
No/yes	0.74	0.46, 1.19	0.212			
Regorafenib initial dose						
80 mg	—	—				
120 mg	0.67	0.35, 1.28	0.23			
160 mg	1.01	0.60, 1.73	0.959			
Locoregional treatment in second‐line treatment						
No/yes	0.77	0.49, 1.21	0.259			
Group						
Regorafenib/regorafenib + ICI	0.56	0.35, 0.88	0.012*	0.55	0.35, 0.87	0.01*

*Note:* Statistical significance indicated in bold (*p*<0.05).

Abbreviations: ALBI, albumin–bilirubin; AFP, α‐fetoprotein; BCLC, Barcelona Clinic Liver Cancer; CI, confidence interval; ECOG, Eastern Cooperative Oncology Group; HBsAg, hepatitis B surface antigen; HR, hazard ratio; ICI, immune checkpoint inhibitors.

### 3.4 Subgroup analysis

**FIGURE 4:**
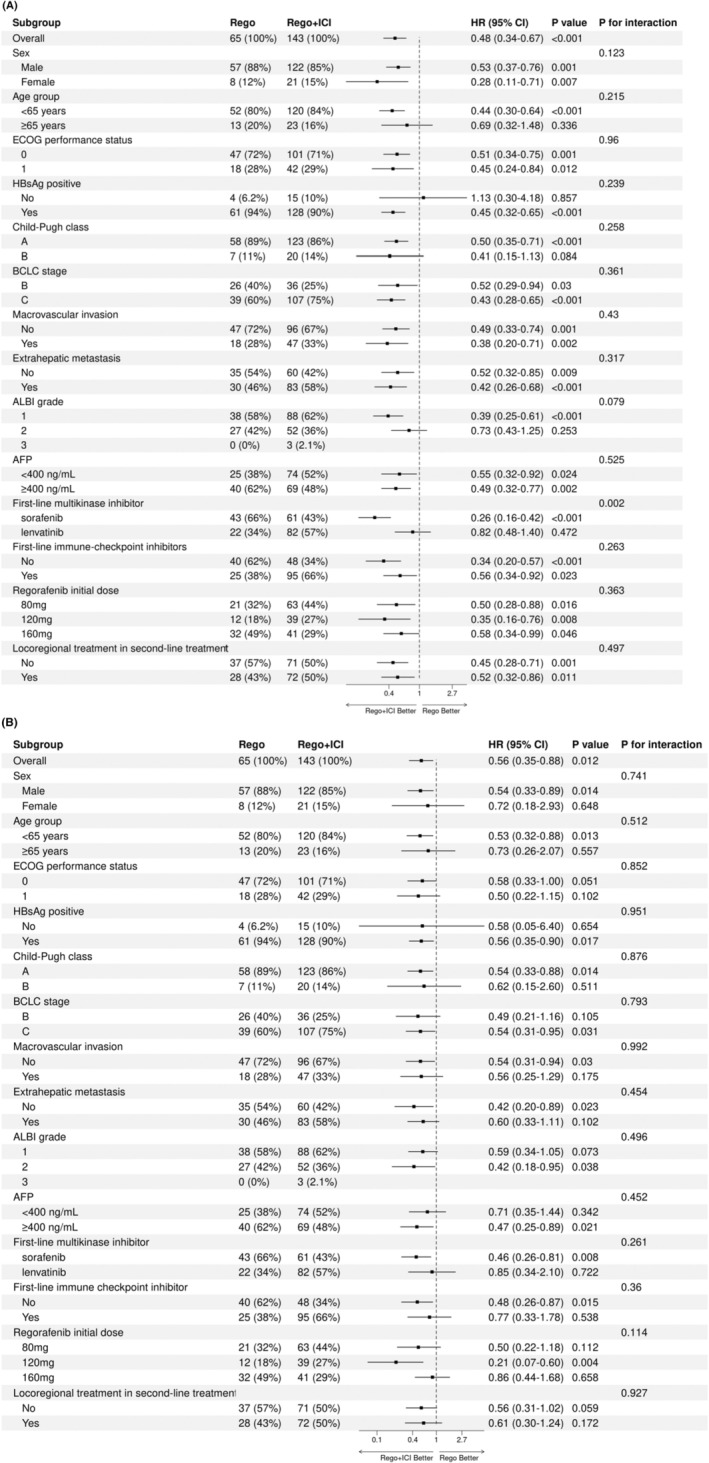
Subgroup analyses for comparing progression‐free survival (A) and overall survival (B) between regorafenib plus ICI group (Rego + ICI) and regorafenib monotherapy group (Rego). AFP, α‐fetoprotein; ALBI, albumin‐bilirubin; BCLC, Barcelona Clinic Liver Cancer; CI, confidence interval; ECOG, Eastern Cooperative Oncology Group; HR, hazard ratio; HBsAg, hepatitis B surface antigen.

Subgroup analyses were conducted according to different clinical characteristics and treatment options. The improvement in PFS with the regorafenib plus ICI group was maintained in nearly all subgroup analyses except for patients with HBsAg‐negative. Patients who received sorafenib in their first‐line treatment (HR = 0.26, 95% CI: 0.16–0.42) had better PFS compared to patients who received lenvatinib (HR = 0.82, 95% CI: 0.48–1.40; *p* for interaction = 0.002; Figure 4A). The improvement in OS with the regorafenib plus ICI group was observed in all subgroup analyses and there were no statistically significant interactions within subgroups (Figure 4B).

### 3.5 Safety

No treatment‐related death happened. The incidence of TRAEs was similar in both groups, with 106 (74.1%) patients who received regorafenib plus ICI and 46 (70.8%) patients who received regorafenib having at least one TRAE. The most common TRAEs of any grade in regorafenib plus ICI group were hand–foot skin reaction (39.2%), diarrhea (29.4%), fatigue (21.7%), increased blood bilirubin (20.3%), and in regorafenib monotherapy group were hand–foot skin reaction (35.4%), diarrhea (24.6%), and increased blood bilirubin (23.1%) (Table 5).

**TABLE 5:** Treatment‐related adverse events.

Treatment‐related adverse events	Regorafenib + ICI (*N* = 143)		Regorafenib (*N* = 65)	
	Any grade (%)	Grade ≥3 (%)	Any grade (%)	Grade ≥3 (%)
Any adverse event	106 (74.1)	34 (23.8)	46 (70.8)	13 (20.0)
Hand–foot skin reaction	56 (39.2)	8 (5.6)	23 (35.4)	3 (4.6)
Diarrhea	42 (29.4)	5 (3.5)	16 (24.6)	2 (3.1)
Fatigue	31 (21.7)	1 (0.7)	9 (13.8)	0 (0)
Hypertension	25 (17.5)	3 (2.1)	6 (9.2)	0 (0)
Increased blood bilirubin	29 (20.3)	5 (3.5)	15 (23.1)	3 (4.6)
Increased AST	20 (14.0)	3 (2.1)	12 (18.5)	1 (1.5)
Increased ALT	19 (13.3)	3 (2.1)	11 (16.9)	0 (0)
Hypoalbuminemia	22 (15.4)	4 (2.8)	6 (9.2)	0 (0)
Thrombocytopenia	20 (14.0)	3 (2.1)	5 (7.7)	1 (1.5)
Hypothyroidism	18 (12.6)	0 (0)	8 (12.3)	0 (0)

*Note*: Data are presented as number (percentage).

Abbreviations: AST, aspartate transaminase; ALT, alanine transaminase; ICI, immune checkpoint inhibitors.

## 4 DISCUSSION

In this multicenter, real‐world, retrospective study, which is one of the largest scale conducted so far, the regorafenib plus ICI group demonstrated significantly higher ORR and DCR compared to the regorafenib monotherapy group based on mRECIST criteria. Median PFS and OS were also significantly improved in the regorafenib plus ICI group. Importantly, combining with ICI did not result in an increased incidence of TRAEs.

The median PFS in the regorafenib monotherapy group (3.2 months) was consistent with the REFINE study (3.9 months). The median OS in the regorafenib monotherapy group (16.4 months) was longer than the overall population in the REFINE study (13.2 months), but was similar to patients from Asia (15.0 months) and patients with Child‐Pugh A(15.5 months).^22^ Together with the REFINE study, our study once again confirmed the efficacy of regorafenib as second‐line therapy for advanced HCC in real‐world settings. In our real‐world study, physicians would employ locoregional treatment modalities, including transarterial chemoembolization (TACE) and hepatic arterial infusion chemotherapy (HAIC), after considering liver function and tumor burden. Therefore, the improvement in OS may be partially attributed to the combined usage of locoregional treatments. However, further validation is required through prospective randomized controlled trials.

The efficacy and survival outcomes of the regorafenib plus ICI group in this study aligned with previous studies, demonstrating superiority over the regorafenib monotherapy group.^23^, ^24^, ^25^, ^26^ Remarkably, in this study, the combination of regorafenib and ICI achieved a median OS of 25.6 months, making it one of the few studies in the second‐line treatment for HCC to surpass the 2‐year milestone. Due to the differences in study design and baseline characteristics, the results from different studies cannot be directly compared. In comparison to previous studies, the patients in this study had relatively better liver function, with the majority being classified as Child‐Pugh A, which could independently influence the survival of patients with HCC.^27^ Additionally, the proportion of patients with extrahepatic metastasis and macrovascular invasion was relatively lower, which might also have a positive impact on survival.^28^

It is worth noting that in multivariate and subgroup analyses, patients who received sorafenib in their first‐line treatment had better PFS in the second‐line treatment compared to patients who received lenvatinib. This may be due to the different molecular target spectrums of sorafenib and lenvatinib, suggesting that the choice of first‐line treatment could have an impact on the effectiveness of subsequent therapies. This highlights the importance of considering previous treatment history in treatment decision‐making for advanced HCC. Previous retrospective studies also showed a difference in the survival outcomes of patients receiving regorafenib after different first‐line systemic treatments.^29^ However, it should be noted that the approval of sorafenib occurred earlier than lenvatinib, resulting in differences in the initiation of actual clinical use between the two subgroups. This disparity in exposure time had an impact on the censored rates (sorafenib 52.9% vs. lenvatinib 73.1%, *p* < 0.003) in the second‐line treatment and consequently influenced the results. As more patients have the opportunity to receive second‐line systemic treatment, incorporating patients' first‐line treatment history or taking the first and second‐line treatments as a whole in future study designs may yield more comprehensive and insightful results and eventually prolong survival.

Implementation of ICI may have collateral effects on the immune system, potentially leading to immune‐related adverse events (irAEs), which could be further amplified by combination strategies.^30^ The safety profile observed in our study somewhat alleviated this concern, as the combination of regorafenib and ICI did not result in a significantly increased incidence of TRAEs or new TRAEs, which is consistent with previous retrospective studies.^23^, ^24^, ^25^, ^26^ The overall incidence of TRAEs was lower compared to the RESORCE trial, where all patients (374 out of 374) who received regorafenib experienced at least one TRAE.^8^ Apart from differences in initial regorafenib dose, this may also be attributed to more extensive clinical experience and more sophisticated management of AEs.

This study had several limitations. First, different PD‐1/PD‐L1 inhibitors applied in the regorafenib plus ICI group may impact the results. Second, this study was conducted in China, with most patients having HBV infection. Further validation in global multicenters with diverse populations is needed. Third, this study did not assess whether the combination of regorafenib and ICI can provide survival benefits after the failure of atezolizumab plus bevacizumab or sintilimab plus bevacizumab. It would be valuable to explore treatment strategies following the progression of these newly emerged combinations.

## 5 CONCLUSIONS

The combination of regorafenib and ICI shows potential as a viable second‐line treatment for advanced HCC, demonstrating favorable efficacy with a manageable safety profile compared to regorafenib monotherapy under real‐world circumstances. Furthermore, as this study included a broader patient population, the survival benefit of regorafenib combined with ICI was validated in patients who progressed on sorafenib or lenvatinib, augmenting the practicality of implementing this treatment regimen in clinical practice.

## AUTHOR CONTRIBUTIONS

**Liang Qiao:** Data curation (equal); formal analysis (equal); writing – original draft (equal). **Wei He:** Data curation (equal); formal analysis (equal); writing – original draft (equal). **Guoying Wang:** Data curation (equal); formal analysis (equal); writing – review and editing (equal). **Huanwei Chen:** Data curation (equal); formal analysis (equal); writing – review and editing (equal). **Fuxi Huang:** Data curation (equal); formal analysis (equal); writing – review and editing (equal). **Bo Zhang:** Data curation (equal); formal analysis (equal); writing – review and editing (equal). **Yuxiong Qiu:** Writing – original draft (supporting). **Shaoru Liu:** Writing – original draft (supporting). **Zhenkun Huang:** Writing – original draft (supporting). **Yichuan Yuan:** Writing – original draft (supporting). **Jiliang Qiu:** Writing – review and editing (supporting). **Yunfei Yuan:** Conceptualization (equal); supervision (equal). **Binkui Li:** Conceptualization (equal); supervision (equal).

## FUNDING INFORMATION

This research did not receive any specific grant from funding agencies in the public, commercial, or not‐for‐profit sectors.

## CONFLICT OF INTEREST STATEMENT

The authors declare no financial interests or personal relationships which may be considered as potential competing interests in this work.

## Data Availability

The data that support the findings of this study are available on request from the corresponding author. The data are not publicly available because of privacy or ethical restrictions.
